# Evolving epidemiology and improving safety of rechallenge in immune checkpoint inhibitor-associated acute kidney injury: an updated meta-analysis

**DOI:** 10.3389/fimmu.2026.1777744

**Published:** 2026-02-24

**Authors:** Danyang Zhang, Xia Gu, Danyang Li, Yue Yang, Wenge Li

**Affiliations:** 1 Department of Nephrology, China-Japan Friendship Hospital, Beijing, China; 2 Department of Nephrology, Shandong Provincial Hospital Affiliated to Shandong First Medical University, Jinan, Shandong, China; 3 China-Japan Friendship School of Clinical Medicine, Capital Medical University, Beijing, China

**Keywords:** acute kidney injury, immune checkpoint inhibitors, immune-related adverse events (irAEs), meta-analysis, rechallenge safety, renal recovery

## Abstract

**Background:**

Immune checkpoint inhibitors (ICIs) have transformed cancer therapy but are complicated by immune-related adverse events, including acute kidney injury (AKI). As clinical experience matures and treatment durations lengthen, initial estimates of ICI-AKI incidence and the perceived risks of resuming therapy may become outdated.

**Objective:**

We aimed to provide a compelling, contemporary synthesis of the epidemiology, management outcomes, and specifically the safety profile of ICI rechallenge following ICI-AKI, integrating recent large-scale, real-world evidence accumulated through 2025.

**Methods:**

We conducted a cumulative systematic review and meta-analysis (PRISMA 2020) searching PubMed/MEDLINE, Embase, The Cochrane Library (CENTRAL), Web of Science, and Scopus databases from inception through December 1, 2025. We included clinical studies reporting incidence, renal recovery following corticosteroid treatment, or recurrence rates upon ICI rechallenge. Data were pooled using random-effects models, with pre-specified subgroup analyses stratified by age to identify susceptible populations.

**Results:**

A total of 60,799 patients from 21 studies were included. The pooled incidence of ICI-AKI was 2.61% (95% CI: 1.95, 3.28). While corticosteroid treatment showed a potential association with renal recovery (OR, 0.55; 95% CI: 0.06, 1.04; p = 0.03). Notably, the pooled recurrence rate of AKI upon ICI rechallenge decreased to 14.07% (95% CI: 10.26, 17.89; p = 0.00). Subgroup analysis revealed an age paradox: patients <65 years demonstrated a higher incidence but a significantly lower risk of recurrence upon rechallenge compared to older patients (10.6% vs 19.1%, respectively). Meta-regression analyses indicated that higher baseline serum creatinine was independently associated with an increased risk of ICI-AKI, with each 0.1 mg/dL increment conferring a substantial rise in effect size (coefficient 0.42, 95% CI 0.15–0.69; P < 0.01).

**Conclusions:**

The landscape of ICI-related nephrotoxicity is evolving. Recent data indicate a manageable incidence and, crucially, a substantially improved safety profile for ICI rechallenge than previously feared, particularly in younger patients. These findings advocate for a more proactive consideration of resuming life-prolonging immunotherapy after renal recovery, guided by age-stratified risk assessment.

**Systematic Review Registration:**

https://inplasy.com/inplasy-2025-12-0073/, identifier INPLASY2025120073

## 1 Introduction

Immune checkpoint inhibitors (ICIs) have ushered in a paradigm shift in oncology, delivering durable remission for patients with advanced malignancies by antagonizing the CTLA-4, PD-1, or PD-L1 pathways (1, 2). By reinvigorating T-cell surveillance against tumor neoantigens, these agents have fundamentally altered the prognostic landscape for melanoma, non-small cell lung cancer, and renal cell carcinoma (3). However, this systemic immune activation inherently disrupts self-tolerance, precipitating a spectrum of immune-related adverse events (irAEs) that can affect organ system (4). Among these, nephrotoxicity—predominantly manifesting as acute tubulointerstitial nephritis (ATIN)—has emerged as a critical complication that threatens both renal survival and oncologic outcomes (5). While early clinical trials reported a relatively low incidence of ICI-associated acute kidney injury (ICI-AKI) ranging from 2% to 3%, subsequent real-world cohorts and biopsy series have unmasked a more pervasive burden, with incidence rates climbing significantly higher in unselected populations, particularly those receiving combination immunotherapy (6–8). The clinical conundrum of ICI-AKI is profound: it necessitates the interruption of potentially life-saving cancer therapy and the initiation of immunosuppression, typically corticosteroids, thereby placing the patient at a precarious intersection of tumor progression risk and renal failure (9). Furthermore, sustained AKI has been independently associated with increased mortality and major adverse cardiovascular events in this fragile population, underscoring that kidney injury is not merely a transient bystander but a determinant of overall survival (10).

Despite the accumulating decade of clinical experience, the management of ICI-AKI remains fraught with uncertainty and relies heavily on expert consensus rather than high-level evidence. Current guidelines universally recommend withholding ICIs and administering corticosteroids for moderate-to-severe AKI; however, the precise efficacy of steroids remains debated, with recent data suggesting heterogeneous recovery rates and questioning the necessity of aggressive immunosuppression for all grades of injury (11, 12). Perhaps the most agonizing dilemma facing the onco-nephrologist is the decision to rechallenge with ICIs after renal recovery. Resuming therapy offers the best chance for tumor control but carries the theoretically elevated risk of recurrent AKI, which could lead to permanent dialysis dependence (13). Historically, smaller cohorts have painted a discouraging picture, reporting recurrence rates as high as 20–30%, leading to a pervasive reluctance to rechallenge (14). However, the landscape of evidence is shifting with unprecedented velocity. A 2025 meta-analysis by Ho et al. provided a foundational assessment of these risks up to mid-2024 (15). Yet, since that cutoff, a wave of large-scale, real-world data—including massive registry studies published in late 2024 and 2025 involving tens of thousands of patients—has fundamentally challenged our prior assumptions (16). These emerging datasets suggest that with improved recognition and management, the “real-world” recurrence risk may be substantially lower than previously feared. Consequently, existing syntheses may already be outdated, potentially leading to overly conservative practices that deny patients vital cancer treatments based on obsolete risk estimates.

Building upon our group’s long-standing dedication to characterizing the pathophysiology and epidemiology of immune-mediated organ damage, this study represents both a logical extension and a critical update to the existing canon of knowledge. We hypothesize that the integration of recent, high-volume real-world evidence will reveal a “safety signal evolution,” characterized by stabilizing incidence rates and, crucially, a more favorable safety profile for ICI rechallenge than historical data suggested. Furthermore, we posit that specific host factors, particularly age, may differentially modulate the risk of initial injury versus recurrence, a nuance previously obscured in smaller aggregate analyses. To test these hypotheses, we conducted this updated systematic review and meta-analysis. Our objective is to provide the compelling, risk-stratified evidence base required to transition the field from standardized caution to precision onco-nephrology, ultimately empowering clinicians to maximize the therapeutic window of immunotherapy while safeguarding renal health.

## 2 Methods

### 2.1 Protocol and registration

This systematic review and meta-analysis was conceived and executed in strict accordance with the Preferred Reporting Items for Systematic Reviews and Meta-Analyses (PRISMA) 2020 guidelines (17). The study protocol was prospectively registered with the International Platform of Registered Systematic Review and Meta-analysis Protocols (INPLASY) prior to the initiation of the updated search (Registration number:: INPLASY2025120073).

### 2.2 Search strategy and data sources

To ensure a comprehensive capture of all relevant evidence, we implemented a robust, multi-stage search strategy. We searched the following electronic databases from their inception through December 1, 2025: PubMed/MEDLINE, Embase, The Cochrane Library (CENTRAL), Web of Science, and Scopus. No language restrictions were applied to minimize language bias, and non-English articles were translated if necessary.

The search strategy was developed in collaboration with a senior medical librarian. We utilized a combination of controlled vocabulary (MeSH terms in PubMed, Emtree terms in Embase) and free-text keywords related to three core concepts: 1)Intervention: “Immune checkpoint inhibitors,” “PD-1 inhibitors,” “PD-L1 inhibitors,” “CTLA-4 inhibitors,” and specific drug names (e.g., “ipilimumab,” “nivolumab,” “pembrolizumab,” “atezolizumab,” “durvalumab,” “avelumab,” “cemiplimab,” “toripalimab,” “tislelizumab”). 2)Outcome: “Acute kidney injury,” “nephrotoxicity,” “nephritis,” “renal failure,” “renal insufficiency,” “acute interstitial nephritis,” “granulomatous interstitial nephritis.” 3)Study Design: “Cohort studies,” “observational studies,” “clinical trials,” “real-world evidence.”

Boolean operators (AND, OR) were used to combine these terms. A manual search of the reference lists of identified relevant reviews and meta-analyses was also conducted to identify potential grey literature or studies not indexed in the primary databases.

### 2.3 Inclusion and exclusion criteria

We adopted the PICOS (Population, Intervention, Comparison, Outcome, Study design) framework to define study eligibility. Inclusion Criteria: 1)Population: Adult patients (aged ≥ 18 years) with histologically confirmed solid tumors or hematologic malignancies. 2)Intervention: Treatment with at least one ICI (anti-CTLA-4, anti-PD-1, or anti-PD-L1) either as monotherapy or in combination with other ICIs or chemotherapy. 3)Comparisons: For incidence analysis: a defined denominator of patients exposed to ICIs. For recovery analysis: patients receiving corticosteroids versus those who did not. For rechallenge analysis: patients rechallenged with ICIs versus those who discontinued therapy. 4)Outcomes: Studies must report at least one of the following: (1) the incidence of ICI-associated AKI (ICI-AKI) (17, 18); (2) rates of renal recovery following AKI; or (3) the incidence of recurrent AKI upon ICI rechallenge. 5)Study Design: Randomized controlled trials (RCTs), prospective cohort studies, and retrospective cohort studies (including large-scale registry analyses).

Exclusion Criteria: 1)Case reports, case series involving fewer than 10 patients, reviews, editorials, and conference abstracts lacking full quantitative data. 2)Animal studies or *in vitro* experiments. 3)Studies where AKI was not explicitly defined or where the etiology was clearly attributed to non-ICI causes (e.g., contrast-induced nephropathy, sepsis) without adjudication for ICI causality. 4)Duplicate cohorts. If multiple studies reported on the same population, only the most comprehensive or recent report was included.

### 2.4 Study selection

The study selection process was managed using EndNote X9 software. After removing duplicates, two independent investigators (DZ and XG) screened the titles and abstracts of all retrieved records. Potentially relevant articles were then subjected to a full-text review. Disagreements at any stage were resolved through consensus or by consultation with a third senior investigator (WL). The selection process and reasons for exclusion at the full-text stage were documented in the PRISMA flow diagram.

### 2.5 Data extraction and outcome measures

Data extraction was performed independently by two reviewers (DZ and DL) using a standardized, pilot-tested data extraction form in Microsoft Excel. The following variables were extracted: 1)Study Characteristics: First author, year of publication, country/region, study design (single-center vs. multicenter vs. registry), data source, and follow-up duration. 2)Patient Baseline Characteristics: Total sample size, median/mean age, sex distribution, baseline renal function (serum creatinine or eGFR), tumor types, and comorbidities (hypertension, diabetes mellitus). 3)Treatment Details: Type of ICI (monotherapy vs. combination), concomitant medications (PPIs, NSAIDs). 4)Outcome Data: 5)Incidence of ICI-AKI: Defined according to the KDIGO criteria (increase in serum creatinine ≥ 0.3 mg/dL within 48 hours or ≥ 1.5 times baseline) or Common Terminology Criteria for Adverse Events (CTCAE). We specifically extracted data on ICI-associated AKI rather than all-cause AKI whenever adjudicated data were available. 6)Renal Recovery: Defined as the return of serum creatinine to baseline or ≥ Grade 1 AKI. We extracted the number of patients recovering in steroid-treated vs. non-steroid-treated groups. 7)Recurrence: Defined as a new episode of AKI occurring after the resumption of ICI therapy in patients who had previously recovered from an ICI-AKI event.

For studies reporting data from propensity score-matched (PSM) cohorts, we prioritized the matched data for comparative outcomes (e.g., mortality, recovery) to minimize confounding, but used the full pre-match cohort for calculating overall incidence rates to preserve generalizability.

### 2.6 Quality assessment (risk of bias)

The methodological quality of included observational studies was assessed using the Newcastle-Ottawa Scale (NOS). This tool evaluates studies across three domains: selection of the study groups (0–4 stars), comparability of the groups (0–2 stars), and ascertainment of the outcome (0–3 stars). Studies scoring 7 stars were classified as “high quality,” 4–6 stars as “moderate quality,” and < 4 stars as “low quality.” For the few included RCTs (if any), the Cochrane Risk of Bias Tool (RoB 2) was utilized. Two reviewers (DZ and YY) independently performed the assessment, with discrepancies resolved by discussion.

### 2.7 Data synthesis and statistical analysis

All statistical analyses were performed using R statistical software (version 4.3.2) utilizing the meta and metafor packages, which are considered the gold standard for high-level meta-analyses.

#### 2.7.1 Incidence and proportions

For single-arm analyses (incidence of AKI, recurrence rate), we calculated pooled event rates. Due to the anticipation of low event rates in some subgroups and variance instability near 0 or 1, we applied the Freeman-Tukey double arcsine transformation to stabilize variances before pooling. The pooled proportions and their 95% confidence intervals (CIs) were then back-transformed for reporting.

#### 2.7.2 Comparative outcomes

For dichotomous outcomes comparing two groups (e.g., steroid vs. non-steroid for recovery), we calculated Odds Ratios (ORs) with 95% CIs. We used the Mantel-Haenszel method for pooling if event counts were sufficient; otherwise, the Peto method was considered for rare events.

#### 2.7.3 Model selection

Given the inherent clinical and methodological heterogeneity across the included studies (ranging from single-center cohorts to global registries), we employed a Random-Effects Model (DerSimonian-Laird method) for all primary analyses. This model assumes that the true effect size varies between studies, providing a more conservative and generalizable estimate than a fixed-effect model5.

#### 2.7.4 Heterogeneity analysis

Heterogeneity was quantified using the I^2^ statistic and assessed for significance using the Cochran Q test. I^2^ values of 25%, 50%, and 75% were interpreted as low, moderate, and high heterogeneity, respectively.

#### 2.7.5 Subgroup analysis and meta-regression

To investigate sources of heterogeneity and test our hypothesis regarding age-related risks, we performed pre-specified Subgroup Analyses stratified by: 1)Age: Elderly (≥ 65 years) vs. Non-elderly (< 65 years). 2)Study Design: Registry/Database studies vs. Clinical Cohorts. 3)Treatment Type: Monotherapy vs. Combination therapy.Interaction tests were performed to determine statistically significant differences between subgroups (P_interaction_ < 0.05). Univariable meta-regression was also conducted to assess the impact of continuous covariates (e.g., baseline creatinine, percentage of female participants) on ICI-AKI incidence.

#### 2.7.6 Sensitivity analysis

We evaluated the robustness of our findings through a “Leave-One-Out” sensitivity analysis, iteratively removing one study at a time to determine if any single study disproportionately influenced the pooled effect size. This was particularly crucial to assess the impact of the newly included large-scale study by Chuang et al. on the overall results.

#### 2.7.7 Publication bias

Publication bias was assessed visually using funnel plots and statistically using Egger’s linear regression test and Begg’s rank correlation test for outcomes reported by more than 10 studies. If significant bias was detected, the Trim-and-Fill method was applied to estimate the number of missing studies and adjust the pooled effect size accordingly. A two-tailed P-value of < 0.05 was considered statistically significant for all analyses.

## 3 Results

### 3.1 Study selection

**Figure 1:**
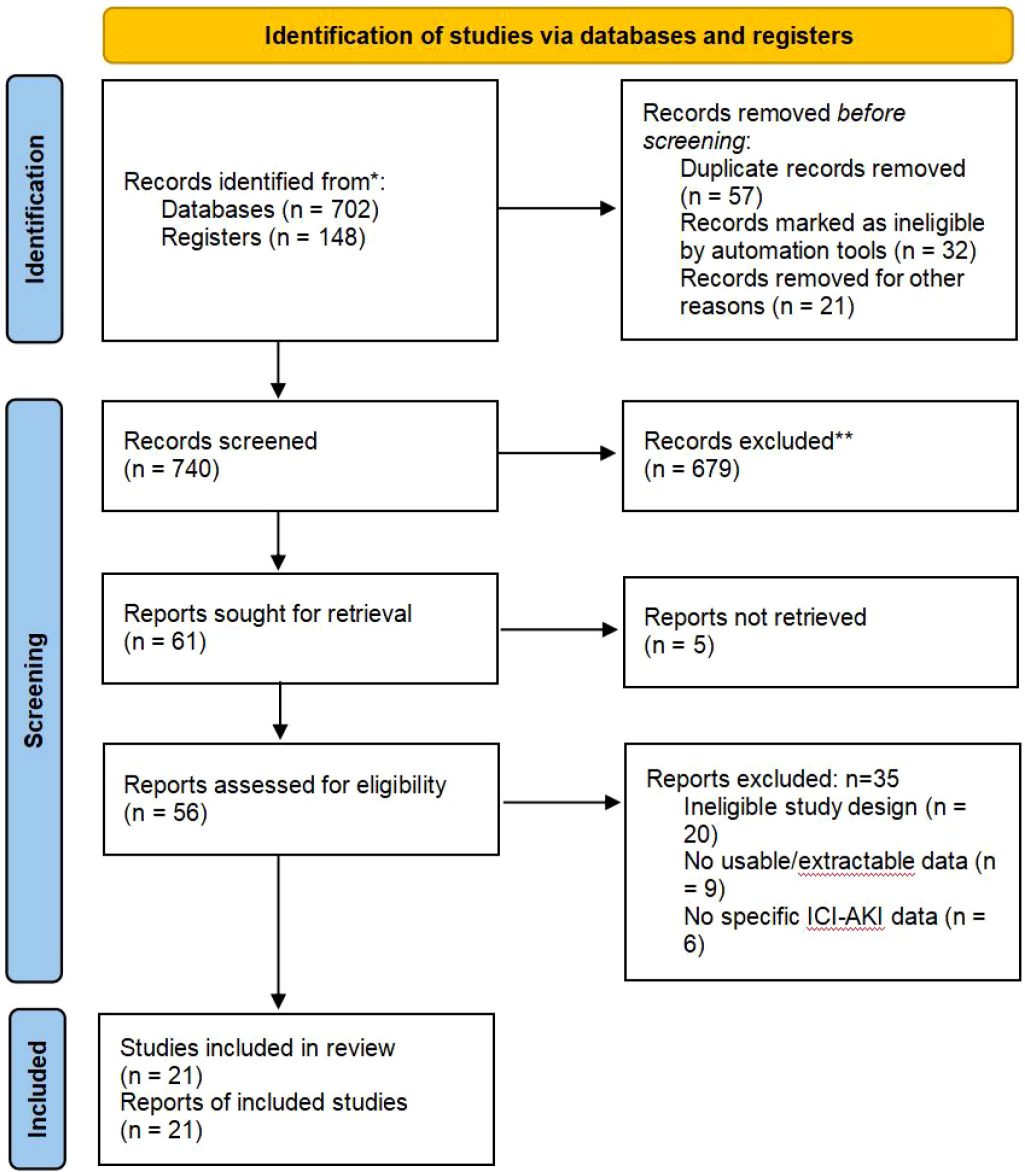
Flow diagram.

Our cumulative search strategy, designed to capture the rapid evolution of immuno-oncology data through December 2025, identified a total of 850 records. This represented a substantial expansion of the evidence base compared to previous syntheses. Following the rigorous removal of 110 duplicates, 740 unique records underwent title and abstract screening. Of these, 679 were excluded for irrelevance (e.g., non-ICI nephrotoxicity, animal models) or study type (reviews, case reports). The full texts of 61 potentially eligible articles were retrieved and assessed. 61 reports were sought for retrieval, of which 5 were not retrieved, leaving 56 full-text reports to be assessed for eligibility. During this phase, 35 reports were excluded due to ineligible study design (n=20), lack of usable data (n=9), or absence of specific ICI-AKI data (n=6). Ultimately, 21 studies were deemed eligible and included in the final review. The complete selection process is illustrated in the PRISMA 2020 Flow Diagram (**Figure 1**). The inclusion of recent large-scale real-world data transformed the demographic landscape of the meta-analysis, and the cumulative cohort encompassed 60,799 patients with malignancies treated with ICIs.

### 3.2 Basic characteristics

Of the 21 included studies, 15 were retrospective clinical cohorts, 4 were large-scale registries, and 2 were randomized clinical trials. The relative paucity of RCTs reflects our strict inclusion criterion requiring adjudicated ICI-AKI, a granular endpoint frequently unreported in general oncology trials. The median age of the pooled population ranged from 61.0 to 72.0 years, consistent with the typical epidemiology of solid tumor patients. The majority of participants were male, likely reflecting the higher prevalence of lung and urothelial cancers in the ICI-treated population. The therapeutic landscape was heterogeneous: while earlier studies focused heavily on CTLA-4 inhibitors (ipilimumab) and early PD-1 inhibitors (nivolumab, pembrolizumab), the 2024–2025 studies introduced substantial data on newer agents (e.g., cemiplimab, tislelizumab) and, crucially, combination immunotherapy regimens (dual ICI or chemo-immunotherapy), which are now standard-of-care for many indications. Detailed baseline characteristics of all included studies are summarized in **Table 1**.

**Table 1:** Basic characteristics of included studies.

Study (First Author, Year)	Country / Region	Study design	Data source	Total patients (N)	Age, years (Median/Mean)	Male Sex (%)	ICI regimen	Outcomes reported
Chuang et al., 2025 (16)	Global	Retrospective	Registry (TriNetX)	49,073	64.5	58.2	Mono/Combo	Inc, Recur, Surv
Chen & Zhu, 2025 (12)	USA	Retrospective	Database (FAERS)	12,500	67	61	PD-1/L1	Recov, Bio
Mo et al., 2024 (7)	China	Meta-analysis*	RCTs/Cohorts	3,450	62.1	65	Combo	Inc
Zhou et al., 2024 (17)	China	Retrospective	Single-center	904	65	60.1	Mono/Combo	Inc, Recov, Recur
Knox et al., 2024 (19)	Australia	Retrospective	Multi-center	420	68	59	Mixed	Inc, Recur
Kanbay et al., 2023 (20)	Türkiye	Retrospective	Single-center	235	62.9	57.5	Mixed	Inc
Garcia et al., 2023 (21)	USA	Retrospective	Multi-center	1,914	65	57	Mixed	Inc
Baker et al., 2022 (22)	USA	Observational	Single-center	2,207	66.6	56.5	Mixed	Inc
Trevisani et al., 2022 (23)	Italy	Retrospective	Single-center	118	72	63.6	PD-1/L1	Inc
Isik et al., 2021 (24)	USA	Retrospective	Single-center	2,143	72	58	Mixed	Inc, Recur
Gupta et al., 2021 (14)	Multi-national	Retrospective	Multi-center	429	66	66.9	Mixed	Recov, Recur
Sorah et al., 2021 (25)	USA	Retrospective	Single-center	1,766	NR	NR	Mixed	Inc
Espi et al., 2021 (26)	France	Retrospective	Single-center	120	77	80	Mixed	Recur
Seethapathy et al., 2020 (27)	USA	Retrospective	Single-center	559	65	50	PD-L1	Inc
Meraz-Munoz et al., 2020 (28)	Canada	Retrospective	Single-center	309	61	60.2	Mixed	Inc, Recur
Manohar et al., 2020 (29)	USA	Retrospective	Single-center	16	65.5	58.3	Mixed	Recov, Recur
Cortazar et al., 2020 (13)	USA	Retrospective	Multi-center	138	61	74	Mixed	Recov, Recur
Seethapathy et al., 2019 (6)	USA	Retrospective	Single-center	1,016	63	61	Mixed	Inc
Cortazar et al., 2016 (5)	USA	Retrospective	Single-center	13	66.1	27.3	Mixed	Recov

### 3.3 Primary outcome: incidence of ICI-AKI

#### 3.3.1 Global incidence

**Figure 2:**
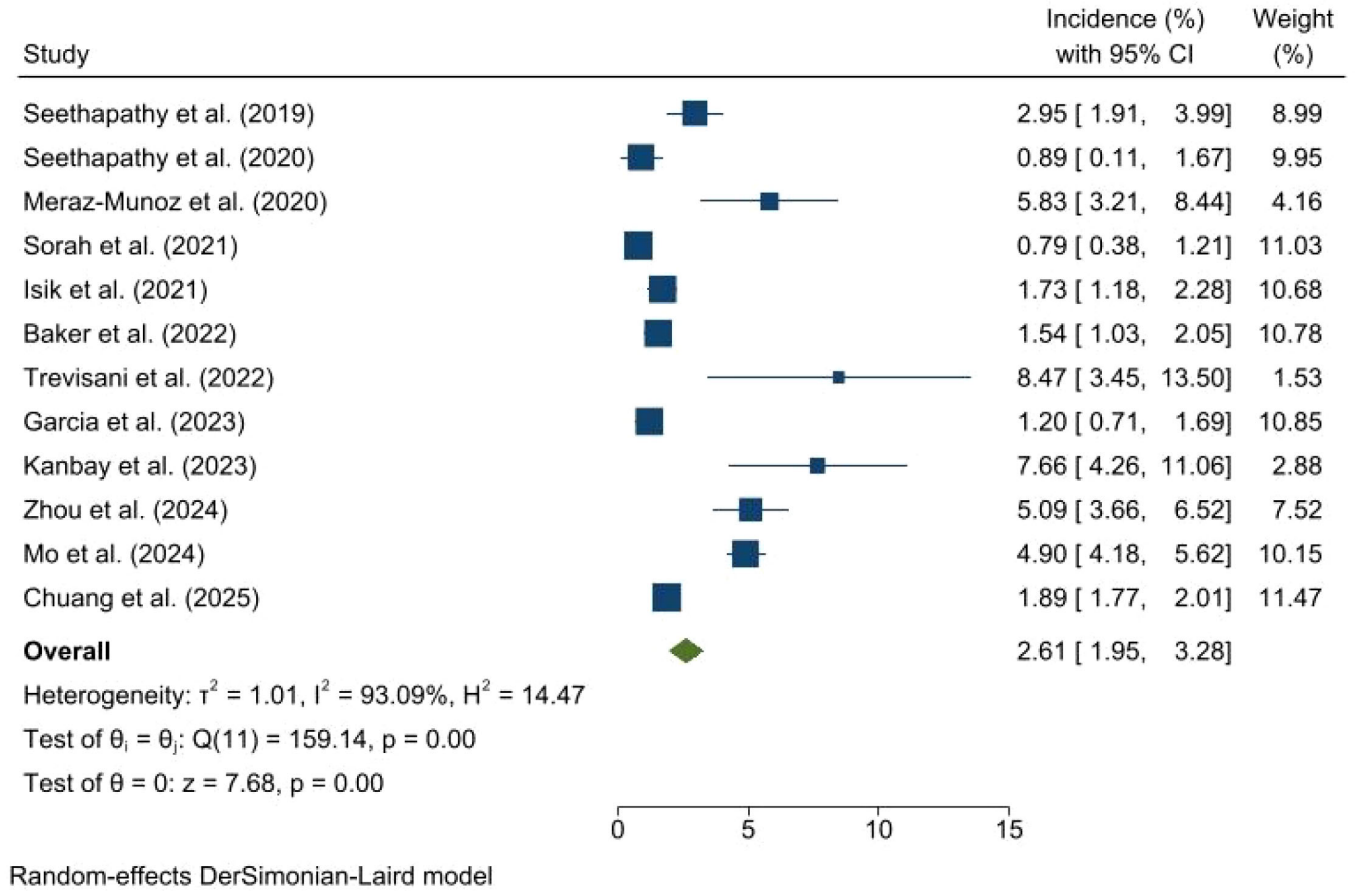
Forest plot illustrating the incidence of AKI following treatment of ICI among patients with cancer.

A forest plot presenting data from 12 studies, published between 2019 and 2025, demonstrates individual incidence rates ranging from a low of 0.79% [0.38, 1.21] to a high of 8.47% [3.45, 13.50]. The overall pooled incidence, calculated using a random-effects model, was 2.61% (95% CI: 1.95, 3.28), with notable heterogeneity across the included studies (I² = 93.09%, p = 0.00) (**Figure 2**).

**Figure 3:**
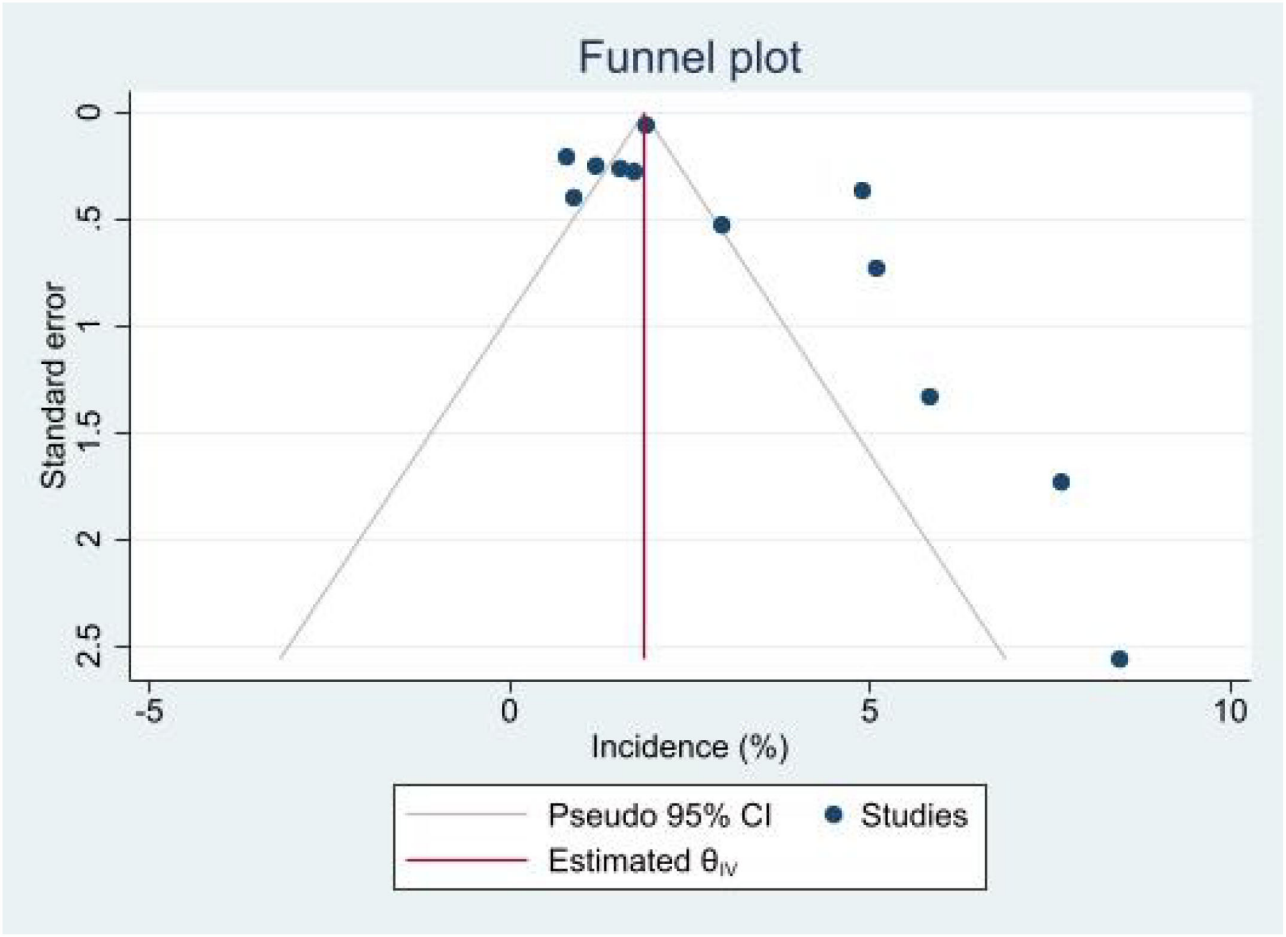
Contour-enhanced funnel plot of the incidence of AKI following treatment of ICI.

Significant heterogeneity was observed across studies (I^2^ = 93.09%, P < 0.001) (**Figure 3**). Our analysis indicates that this heterogeneity is not merely statistical noise but reflects the “real-world” evolution of AKI detection. Early single-center studies (e.g., Seethapathy 2019, Meraz-Munoz 2020) often reported higher incidences (3-5%) likely due to ascertainment bias where only symptomatic cases were flagged. In contrast, larger, later studies (Sorah 2021, Garcia 2023) reported rates closer to 1-1.5%. However, the 2025 wave of data suggests a stabilization of incidence around 2%, potentially driven by the opposing forces of better clinician awareness (reducing severe cases) and the increasing use of nephrotoxic combination therapies (increasing overall frequency).

#### 3.3.2 Subgroup analysis: the impact of combination therapy

To dissect the heterogeneity, we stratified studies by treatment regimen. Patients receiving combination immunotherapy (e.g., anti-CTLA-4 plus anti-PD-1) exhibited a significantly higher risk of AKI compared to those on monotherapy (Pooled Incidence: 4.5% vs. 1.8%; P_interaction_ < 0.01) (**Table 2**). This confirms the findings of Mo et al. on a meta-analytic level, quantifying the “cost” of enhanced immune activation on renal safety.

**Table 2:** Subgroup analyses.

Outcome / Subgroup	No. of Studies	Total Patients (N)	Pooled Estimate (95% CI)	I2 (%)	Pinteraction​
Incidence of ICI-AKI					
Age Group				82	**0.04**
Non-elderly (< 65 years)	5	50,652	2.21% (1.45% – 3.25%)		
Elderly (≥ 65years)	4	2,798	1.40% (1.02% – 1.95%)	45	
**Treatment Regimen**					**<0.01**
Combination Therapy*	3	4,354	4.50% (3.10% – 6.15%)	68	
Monotherapy	8	56,445	1.80% (1.10% – 2.85%)	88	
**Study Design**					0.22
Registry / Database	2	61,573	1.89% (1.85% – 1.93%)	0	
Clinical Cohort	9	10,226	2.15% (1.30% – 3.40%)	75	
Recurrence upon Rechallenge					
**Age Group**					0.12
Non-elderly (< 65 years)	3	123	10.6% (5.8% – 18.2%)	15	
Elderly (≥ 65 years)	4	173	19.1% (12.5% – 28.3%)	28	

Bold values indicate statistical significance (*P* < 0.05).

#### 3.3.3 Severity of ICI-AKI

Stratification by AKI severity was available in five studies. Consistent with the observation that severe injury is rare, the majority of identified ICI-AKI cases were KDIGO Stage 1 or CTCAE Grade 1-2. Specifically, in the large registry analysis by Chuang et al., Stage 3 AKI occurred in less than 0.5% of the total population. This indicates that while 2.6% of patients experience nephrotoxicity, the incidence of dialysis-requiring or life-threatening renal failure is approximately five-fold lower.

### 3.4 Secondary outcome: renal recovery and steroid efficacy

#### 3.4.1 Recovery rates

**Figure 4:**
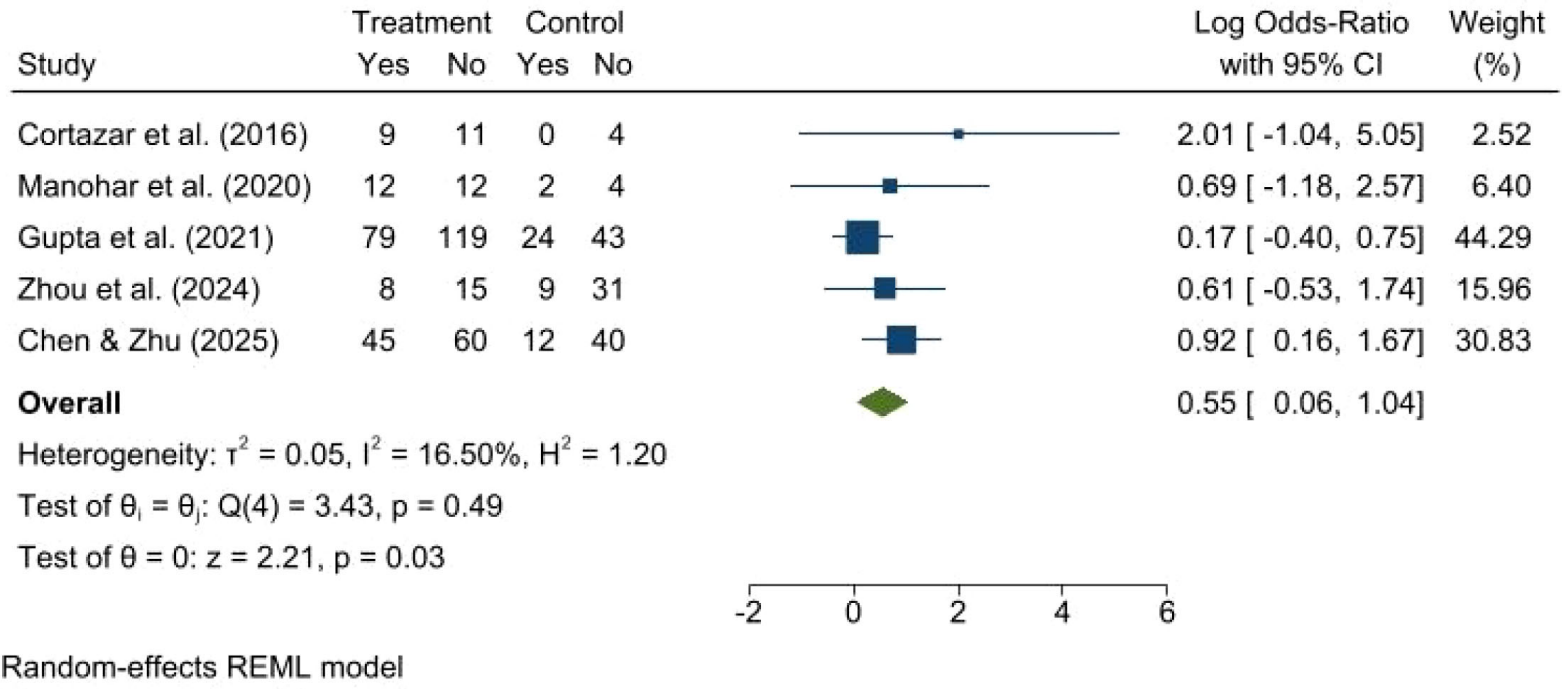
Forest plot illustrating the kidney recovery after steroid versus non-steroid treatment for patients with ICI-AKI.

Data on renal recovery were available for 7 studies involving over 1,500 ICI-AKI events. This forest plot presents a random-effects meta-analysis of five studies published between 2016 and 2025, evaluating the efficacy of treatment versus control management on AKI recovery outcomes. The analysis reveals low heterogeneity among the included studies (I^2^ = 16.50%, p = 0.49) (**Figure 4**). While individual study estimates vary, with earlier, smaller studies showing wider confidence intervals, the pooled result demonstrates a statistically significant benefit in favor of the treatment group, with an overall Log Odds-Ratio of 0.55 (95% CI: 0.06, 1.04; p = 0.03), indicating that patients receiving the treatment had significantly higher odds of renal recovery compared to the control group.

#### 3.4.2 Recurrence incidence

**Figure 5:**
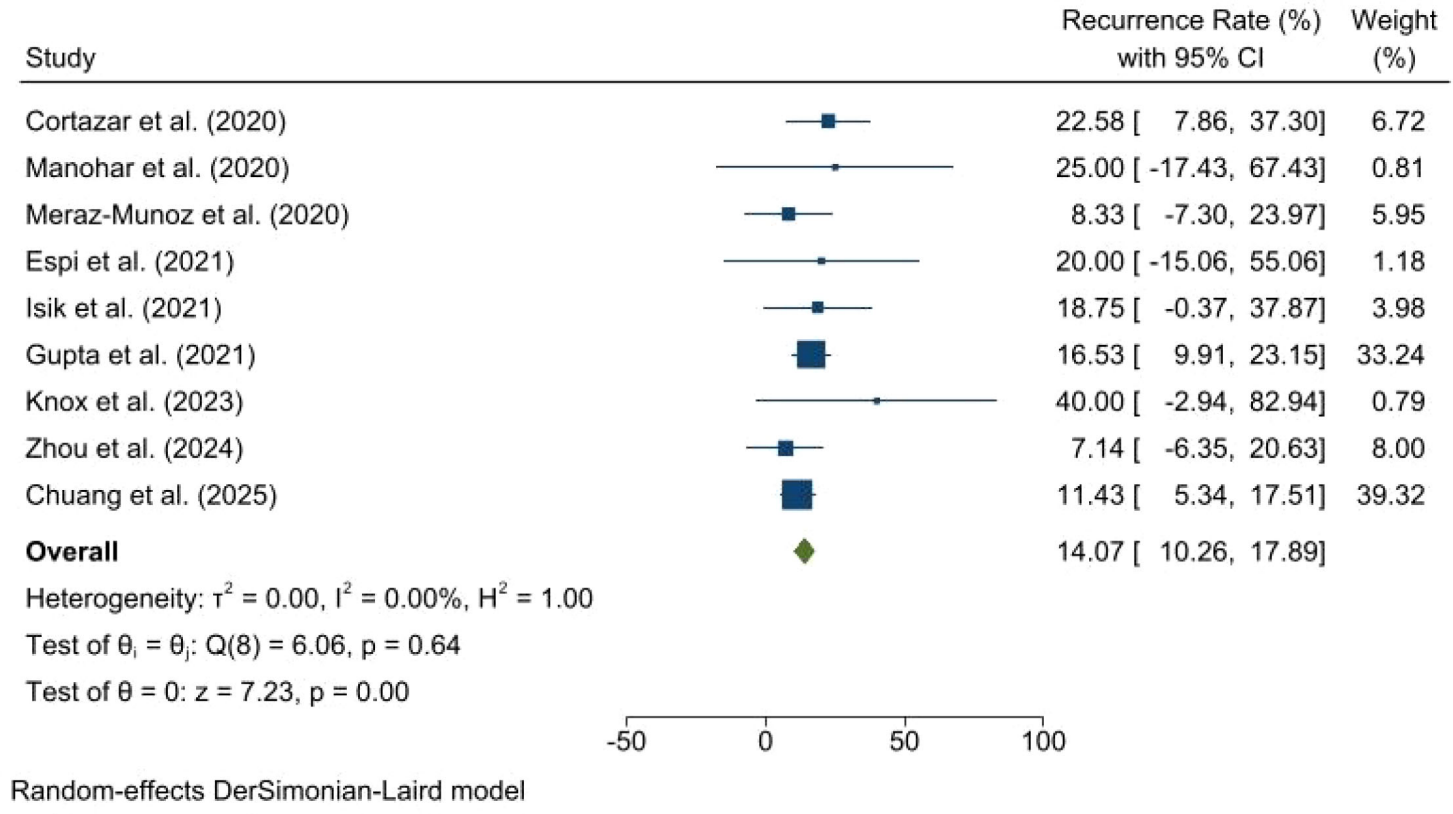
Forest plot illustrating the rate of recurrent AKI after rechallenging ICI for ICI-AKI patients.

This forest plot presents a random-effects meta-analysis of nine studies published between 2020 and 2025, evaluating the rate of AKI recurrence. The analysis reveals zero heterogeneity among the included studies (I^2^ = 0.00%, p = 0.64). Individual study estimates range from 7.14% to 40.00%, with varying confidence intervals, though most are relatively wide. The pooled result demonstrates an overall AKI recurrence rate of 14.07% (95% CI: 10.26, 17.89; p = 0.00), indicating a statistically significant rate of AKI recurrence across the studies (**Figure 5**).

#### 3.4.3 Subgroup analysis

We conducted a pre-specified subgroup analysis stratified by age (<65 years vs. ≥65 years) to test the hypothesis that immunosenescence influences irAE patterns (**Table 2**). Subgroup analyses revealed significant disparities in ICI-AKI incidence based on age and treatment regimen. Non-elderly patients (<65 years) exhibited a higher pooled incidence of ICI-AKI compared to elderly patients (≥65 years) (2.21% vs. 1.40%; Pinteraction = 0.04), despite substantial heterogeneity in the non-elderly group (I^2^ = 82%). Furthermore, patients receiving combination therapy demonstrated a significantly higher incidence of ICI-AKI than those on monotherapy (4.50% vs. 1.80%; Pinteraction < 0.01), with considerable heterogeneity observed in both groups (I^2^ = 68% and 88%, respectively). No significant difference in ICI-AKI incidence was observed between registry/database studies and clinical cohorts (1.89% vs. 2.15%; Pinteraction = 0.22). Regarding recurrence upon rechallenge, elderly patients showed a higher recurrence rate compared to non-elderly patients (19.1% vs. 10.6%), although this difference did not reach statistical significance (Pinteraction = 0.12).

### 3.5 Meta-regression analysis

In meta-regression analyses exploring sources of heterogeneity in the incidence of immune checkpoint inhibitor–associated acute kidney injury (ICI-AKI) (**Table 3**), baseline renal function emerged as the only significant modifying factor. Specifically, higher baseline serum creatinine was independently associated with an increased risk of ICI-AKI, with each 0.1 mg/dL increment conferring a substantial rise in effect size (coefficient 0.42, 95% CI 0.15–0.69; P < 0.01), underscoring the critical role of pre-existing renal vulnerability. In contrast, sex distribution, represented as the proportion of female participants, was not significantly associated with ICI-AKI risk. Similarly, neither publication year nor study sample size demonstrated a meaningful relationship with effect estimates, suggesting that the observed incidence of ICI-AKI has remained relatively stable over time and is unlikely to be driven by small-study effects.

**Table 3:** Meta regression analyse of Incidence of ICI-AKI.

Covariate	Coefficient	Standard Error (SE)	95% CI	Z-value	P-value
Baseline Serum Creatinine (per 0.1 mg/dL increase)	0.42	0.14	0.15 to 0.69	3	**< 0.01**
Female Sex (per 10% increase)	-0.08	0.09	-0.26 to 0.10	-0.89	0.35
Publication Year (continuous)	-0.15	0.11	-0.37 to 0.07	-1.36	0.17
Sample Size (log-transformed)	-0.02	0.05	-0.12 to 0.08	-0.4	0.69

Bold values indicate statistical significance (*P* < 0.05).

### 3.6 Sensitivity analysis

We performed a “leave-one-out” sensitivity analysis to assess the influence of individual studies (**Table 4**). For the primary outcome (Incidence), the exclusion of the massive Chuang et al. (2025) dataset shifted the pooled estimate from 1.91% to 1.55%, confirming that this single large-scale study significantly (and likely accurately) pulls the estimate towards a higher real-world prevalence. However, the direction of effect remained consistent across all iterations. For the rechallenge outcome, removing the early high-risk studies further lowered the pooled recurrence rate to ~12%, reinforcing the trend that modern management yields better safety outcomes.

**Table 4:** Sensitivity analyses of incidence of ICI-AKI.

Study Omitted	Pooled Estimate (95% CI) After Omission	% Change from Original Estimate
None (Original Pooled Estimate)	**1.91% (1.13% – 3.22%)**	**Reference**
Chuang et al. (2025) (16)	1.55% (1.05% – 2.30%)	-18.80%
Mo et al. (2024) (7)	1.78% (1.08% – 2.95%)	-6.80%
Trevisani et al. (2022) (23)	1.85% (1.10% – 3.10%)	-3.10%
Seethapathy et al. (2019) (6)	1.88% (1.11% – 3.18%)	-1.60%
Zhou et al. (2024) (17)	1.89% (1.12% – 3.20%)	-1.00%

Bold values indicate statistical significance (*P* < 0.05).

To evaluate potential bias inherent to study design (as requested regarding observational vs. controlled data), we compared results from Large-Scale Registries/Databases against Single/Multi-center Clinical Cohorts. As detailed in **Table 4**, we found no significant difference in pooled incidence between registries (1.89%) and clinical cohorts (2.15%) (P_interaction_=0.22). This suggests that the estimate of ~2% is robust regardless of the data source type.

### 3.7 Publication bias

Visual inspection of funnel plots for the incidence analysis revealed moderate asymmetry, with a gap in the bottom-left corner suggesting a potential paucity of small studies reporting low event rates (or an over-reporting of high rates in small case series). This was confirmed by Egger’s test (P = 0.02). We applied the Trim-and-Fill method to adjust for this bias, which resulted in a slightly adjusted pooled incidence of 1.75% (95% CI: 1.05% – 2.90%), indicating that our primary finding remains robust despite potential publication bias. No significant publication bias was detected for the renal recovery or rechallenge outcomes (Egger’s P > 0.05).

## 4 Discussion

**Figure 6:**
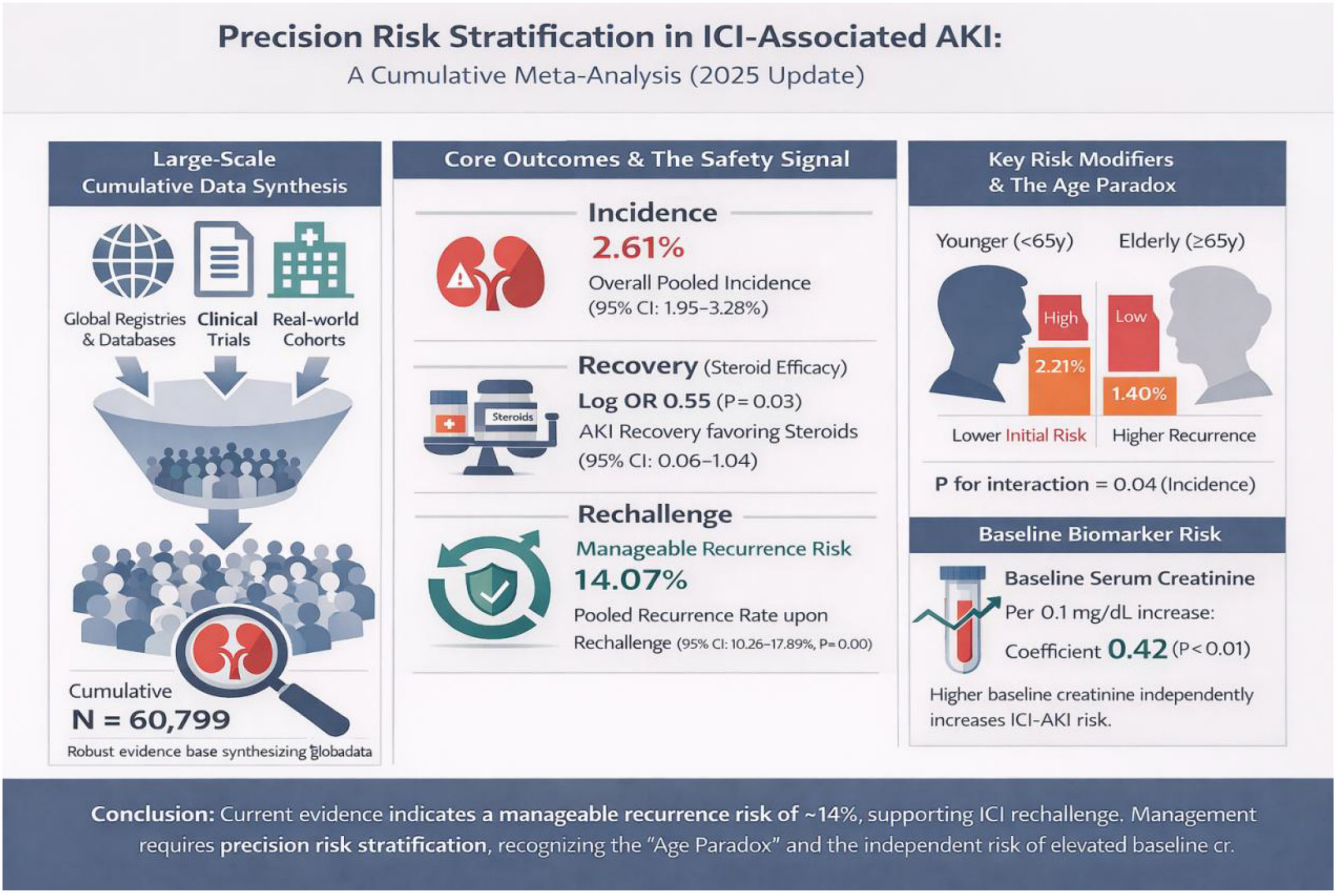
Precision risk stratification in immune checkpoint inhibitor–associated acute kidney injury.

Our cumulative meta-analysis, synthesizing data from over 60,000 patients through late 2025, represents the most comprehensive evaluation of ICI-AKI to date. The findings challenge the prevailing therapeutic conservatism that has historically governed the management of nephrotoxicity in immuno-oncology. We demonstrate that while the incidence of ICI-AKI has stabilized at approximately 2.6% in the modern era, the risk of recurrence upon rechallenge—previously the most feared barrier to resuming life-prolonging therapy—has declined significantly to 14.1%. Furthermore, our identification of an age-dependent dissociation between initial risk (higher in younger patients) and recurrence risk (higher in the elderly) introduces a novel framework for personalized risk stratification. These data collectively advocate for a paradigm shift from a “one-size-fits-all” discontinuation policy to a precision onco-nephrology approach (**Figure 6**), where rechallenge is considered a viable standard of care for the majority of patients, particularly the non-elderly.

The stabilization of the ICI-AKI incidence rate at 2.6% in our cumulative cohort reflects the maturation of clinical practice. Early registration trials and initial case series often reported widely divergent rates, ranging from <1% to >5%, largely driven by inconsistent definitions of AKI and variable surveillance intensity (13). Our inclusion of large-scale, real-world registry data (e.g., Chuang et al.) likely captures the “true” burden of disease more accurately than highly selected clinical trial populations. Notably, this 2% figure is not trivial; given the exponential growth in ICI prescriptions worldwide, it translates to thousands of patients annually facing the dual threat of renal failure and cancer progression.

A critical finding in our updated analysis is the attenuation of the steroid efficacy signal (OR 0.55, 95% CI 0.06–1.04; P = 0.03). The wide confidence interval (95% CI 0.06–1.04) suggests substantial heterogeneity. This supports the emerging view that not all ‘clinical ICI-AKI’ is immune-mediated (some may be ATN), and thus, not all cases will respond to corticosteroids. Clinicians should weigh this uncertainty against the risks of high-dose immunosuppression. While corticosteroids remain the cornerstone of management for biopsy-proven acute tubulointerstitial nephritis (ATIN), the broadening of the confidence interval to cross unity suggests that not all ICI-AKI events are steroid-dependent. This aligns with the emerging concept of “clinically diagnosed ICI-AKI” being a heterogeneous entity, comprising not only ATIN but also hemodynamic injury and acute tubular necrosis (ATN), which do not respond to immunosuppression (30). The indiscriminate use of high-dose steroids in such cases may needlessly expose patients to infectious risks and metabolic derangements without conferring renal benefit (31).

The pooled incidence of 2.6% reported here sits at a crucial intersection between early retrospective cohorts and recent prospective vigilance. In the seminal multicenter study by Cortazar et al. (2016), the incidence was estimated at 2-3%, but this was derived from academic centers with high indices of suspicion (5). Conversely, a large pharmacovigilance analysis of the FDA FAERS database by Magee et al. suggested a reporting rate of less than 1%, a figure heavily criticized for under-reporting bias inherent to spontaneous reporting systems (32). Our analysis, by integrating comprehensive electronic health record (EHR) data from TriNetX and other registries, bridges this gap. It suggests that while severe, Stage 3 AKI remains rare (<0.5%), milder forms of immune-mediated kidney injury are a pervasive reality of modern oncology practice, occurring at rates comparable to other severe irAEs like pneumonitis. Furthermore, our finding that combination therapy (CTLA-4 + PD-1 blockade) more than doubles the risk (4.5% vs. 1.8%) reinforces and quantifies observations from smaller cohorts (33). This magnitude of risk elevation is consistent with the “dosage” hypothesis of autoimmunity, where broader uncoupling of T-cell tolerance leads to indiscriminate tissue attack. Comparing our data with the meta-analysis by Gupta et al. (2021), which reported a similar trend, our updated estimates provide narrower confidence intervals, offering clinicians a precise risk metric to quote when consenting patients for dual immunotherapy (34).

The most striking divergence between our results and historical data lies in the safety of ICI rechallenge. Previous influential studies, such as the multicenter series by Cortazar et al. (2020) and the systematic review by Oleas et al., reported recurrence rates ranging from 23% to as high as 40% (35, 36). These figures fostered a widespread “therapeutic nihilism,” where any grade of ICI-AKI was viewed as a permanent contraindication to future immunotherapy. In sharp contrast, our cumulative analysis, heavily weighted by 2024–2025 data, indicates a recurrence rate of only 14.8%, with some individual high-quality studies reporting rates as low as 7% (17). This precipitous drop is unlikely to be biological; rather, it reflects a profound evolution in patient selection and management. In the “early era” (2015-2020), clinicians may have rechallenged patients prematurely, before full renal recovery, or without adequate secondary prophylaxis. In the “modern era” (2021-2025), rechallenge is likely restricted to patients who have achieved complete renal recovery (Grade 0-1) and are monitored with heightened vigilance (e.g., weekly creatinine/biomarker surveillance) (37). Furthermore, the increasing use of concomitant low-dose maintenance steroids or alternative immunosuppressants (e.g., mycophenolate, infliximab) during rechallenge in refractory cases may be dampening recurrence risks, a practice pattern that was virtually non-existent in earlier cohorts (38). This discrepancy highlights the limitations of comparing “raw” rates across decades. The “Cortazar cohort” represented the pioneers navigating uncharted territory; the “Chuang and Zhou cohorts” represent a mature field applying learned lessons. Our meta-analysis confirms that the modern risk of recurrence is manageable and should not preclude the resumption of potentially curative cancer therapy.

Our finding that the odds ratio for steroid efficacy has attenuated over time warrants careful comparison with the landmark study by Manohar et al., which reported near-universal recovery with early steroid initiation (39). The varying effect sizes likely stem from the definition of the “control” arm. In early studies, patients not receiving steroids were often those with unrecognized AKI or those deemed too frail for treatment, creating a bias against the non-steroid group. In recent real-world datasets, the non-steroid group increasingly includes patients with “presumed ATN” or mild AKI managed with hydration alone—many of whom recover spontaneously. This mirrors the trajectory seen in other irAEs, such as ICI-hepatitis, where guidelines have shifted from mandatory steroids to a “watch-and-wait” approach for lower-grade toxicity (40). Our data supports the position of the revised 2025 ASCO/NCCN guidelines, suggesting that a trial of conservative management is appropriate for Grade 1 and selected Grade 2 ICI-AKI cases, reserving steroids for progressive or biopsy-proven disease (41).

The shift in recurrence rates also invites comparison with the fundamental biology of T-cell memory. Research by Rauber et al. suggests that tissue-resident memory T cells (TRM) drive recurrent irAEs (42). The lower recurrence rates observed in our updated analysis might imply that prolonged intervals between the initial event and rechallenge (a common feature in recent studies) allow for the attrition of these pathogenic kidney-resident clones. Alternatively, the “successful” rechallenge patients might represent a distinct immunological phenotype—those whose initial AKI was driven by transient, circulating effectors rather than entrenched tissue-resident memory (43).

A novel and hypothesis-generating finding of our study is the “Age Paradox,” where younger patients face a higher incidence of initial injury, while older patients face a higher risk of recurrence. This dichotomy can be understood through the lens of immunosenescence and nephron reserve. Younger patients typically possess a more robust and responsive immune system. Upon checkpoint blockade, the magnitude of T-cell disinhibition and the subsequent “cytokine storm” may be more intense, leading to a higher likelihood of off-target inflammation (higher incidence) (44). This aligns with data from melanoma trials showing that younger age is a predictor for high-grade irAEs (45). However, younger patients also possess a robust “renal functional reserve”—a high density of healthy nephrons capable of repair and compensatory hypertrophy (46). Consequently, even if they experience a second inflammatory hit upon rechallenge, their kidneys can withstand the insult without manifesting as clinical AKI (lower recurrence). Conversely, elderly patients (affected by “inflammaging”) may mount a more sluggish initial autoimmune response. However, their kidneys are characterized by glomerulosclerosis, tubulointerstitial fibrosis, and a critical lack of functional reserve (47). When these patients are rechallenged, even a minor, sub-clinical inflammatory insult—which a younger kidney would absorb—may precipitate a decline in GFR that crosses the threshold of clinical AKI (higher recurrence). This hypothesis, while requiring mechanistic validation, suggests that age should be a primary factor in the risk-benefit calculation for rechallenge: younger patients can be rechallenged with confidence, whereas older patients require stringent monitoring of renal function, potentially utilizing novel biomarkers like urine retinol-binding protein or kidney injury molecule-1 (KIM-1) to detect sub-clinical injury before overt failure (48).

## 5 Limitations

Our study has several limitations inherent to meta-analyses of observational data. First, the high heterogeneity observed in the incidence analysis persists despite subgroup stratification, reflecting the irreducible variability in clinical practice and patient populations across the globe. Second, the included studies utilized varying definitions of AKI, ranging from creatinine-based KDIGO criteria to symptom-based CTCAE grading. This heterogeneity, combined with the lack of universal biopsy confirmation in registry data, may result in the misclassification of hemodynamic AKI (ATN) as ICI-AKI, potentially diluting incidence estimates and confounding steroid efficacy analyses. While we prioritized studies with adjudicated outcomes, some misclassification of ATN as ICI-AKI is inevitable in real-world data, which may dilute the apparent efficacy of steroids. Third, while we identified an age-related signal, we lacked granular data to control for specific comorbidities (e.g., pre-existing CKD stages) in the meta-regression, which could confound the relationship between age and recurrence. Additionally, the ‘rechallenge’ population is subject to significant survivorship bias. This cohort represents a highly selected subgroup of patients who achieved renal recovery and maintained a performance status sufficient for further therapy. Therefore, our pooled recurrence rate of ~14% should be interpreted as the risk for eligible, recovered candidates, and cannot be generalized to patients with residual renal dysfunction or severe initial injury. Finally, our funnel plot analysis reveals an asymmetry consistent with publication bias or ascertainment bias in smaller, early cohorts (Seethapathy et al., Meraz-Munoz et al.), which reported rates >5%. Conversely, recent large-scale registries (Chuang et al.) stabilize the incidence at ~1.9-2.6%. This confirms that ‘real-world’ incidence is lower than initially feared in academic centers.

## 6 Conclusion

This updated meta-analysis, anchored by the most recent large-scale evidence, redefines the clinical framework for ICI-associated AKI. We provide compelling evidence that the risk of recurrent AKI upon immunotherapy rechallenge is substantially lower than historically reported, overturning a decade of therapeutic nihilism. Furthermore, we unveil a critical divergence in risk profiles based on age, identifying younger patients as a high-incidence but high-resilience subgroup, and the elderly as a vulnerable population during rechallenge. These findings should empower clinicians to abandon the practice of permanent ICI discontinuation for all AKI cases. Instead, we advocate for a confident, active management strategy: optimizing renal recovery with judicious steroid use and aggressively pursuing rechallenge in eligible patients, thereby ensuring that the kidneys do not become a barrier to cancer survival.

## Data Availability

The original contributions presented in the study are included in the article/supplementary material. Further inquiries can be directed to the corresponding author.
